# Blood inflammatory markers and mortality in the US population: A Health and Retirement Survey (HRS) analysis

**DOI:** 10.1371/journal.pone.0293027

**Published:** 2023-10-16

**Authors:** Attiya Kalair, Matilde Pavan, Naomi Alpert, Saghi Ghaffari, Emanuela Taioli

**Affiliations:** 1 Institute for Translational Epidemiology. Icahn School of Medicine at Mount Sinai, New York, NY, United States of America; 2 Department of Cell, Developmental and Regenerative Biology and Oncological Sciences, Icahn School of Medicine at Mount Sinai, New York, NY, United States of America; 3 Tisch Cancer Institute, Icahn School of Medicine at Mount Sinai, New York, NY, United States of America; Universita Politecnica delle Marche, ITALY

## Abstract

A potential direct correlation between systemic inflammation and physiological aging has been suggested, along with whether there is a higher expression of inflammatory markers in otherwise healthy older adults. Cross-sectional data were extracted from the publicly available 2016 Health and Retirement Survey, a nationally representative survey of older adults in the United States. A subset of participants (n = 9934) consented to a blood draw at the time of recruitment and were measured for high sensitivity C-reactive protein (hs-CRP), Interleukin (IL-6, IL-10, IL-1RA), soluble tumor necrosis factor receptor (sTNFR-1) and transforming growth factor beta 1 (TGF-β1). We included 9,188 participants, representative of 83,939,225 nationally. After adjusting for sex and the number of comorbidities, there remained a significant positive correlation between age and ln (log adjusted) IL-6, and ln sTNFR-1, and a significant inverse correlation between age and ln IL-1RA, ln TGF-β1, and ln hs-CRP. Among the subset of participants who reported none of the available comorbidities (n = 971), there remained an independent correlation of age with ln IL-6 and ln sTNFR-1. After adjusting for age, sex, and number of reported comorbidities, there was a statistically significant correlation between increased ln IL-6, ln IL-10, ln sTNFR-1, and ln hs-CRP with death. This study highlights the existence of a correlation between serum biomarkers of inflammation and aging, not only in the whole population, but also in the smaller subset who reported no comorbidities, confirming the existence of a presence of low-grade inflammation in aging, even in healthy elders. We also highlight the existence of a correlation between inflammatory markers and overall mortality. Future studies should address a possible threshold of systemic inflammation where mortality significantly increases, as well as explore the effectiveness of anti-inflammatory treatments on morbidity and mortality in healthy aging subjects.

## Introduction

Several studies in the literature highlight the existence of an increase in systemic inflammation as age advances [2–6] and suggest that this may be partly associated to the many comorbidities that affect older adults. Either as a result of, or contributor to, morbidity, [1] however at the same time, a direct correlation between systemic inflammation and physiological aging is still being studied along with whether there are higher levels of inflammatory markers in otherwise healthy elders. While increased levels of inflammatory markers such as interleukin 6 (IL-6), tumor necrosis factor (TNF) and interferon (IFN)-y are very common findings in elderly subjects, not many studies disentangle the effect of chronic diseases from the physiological effect of aging.

In 1998, a study [2] was conducted to verify the differences in levels of inflammatory biomarkers between people of different ages; 595 males and 748 females were included, all individuals healthy and affected by chronic conditions, between the age of 20 and 102 years old. After adjusting for cardiovascular risk factors and morbidity, an inverse relation between IL-6 levels and age was found. Additionally, there was a statistically significant positive correlation of age and serum IL6 receptor (sIL-6r). The increase of sIL-6r with age was confirmed with the analysis of a subgroup of healthy younger 51 males and 45 females with low-risk profiles to conclude presence of sIL-6r increases with age.

Using a similar study design, other studies compared the levels of inflammatory biomarkers between younger and older subjects. In 2003 [3], plasma levels of IL-16 and IL-10 were compared between 138 healthy participants of the Swedish longitudinal NONA study (aged between 86 and 94 years old), and 18 healthy Swedish volunteers (aged between 32 and 59 years). The comparison showed a three-fold increase in IL-6 levels in the older group, whereas IL-10 levels were very similar between the two groups. There was no difference between males and females.

Higher levels of IL-6 in older subjects were also found in a 1992 study [4], in which 42 healthy subjects (7 males and 35 females), aged 26 to 75 years, were divided into two groups (ages 26–54 and ages 55–75 years) and compared. The inflammatory cytokine concentration in the blood was found to be higher in older subjects, particularly in the male group.

Differences in cytokines levels with age were also found in-vitro. In particular, one study [5] analyzed the different production of IL-6, TNF a, IFN-y and IL-1B by peripheral mononuclear cells in two different groups of healthy individuals. The mean age of the two groups was 26.8 years and 80.2 years. After 24-72h of stimulation of mononuclear cells IL-6, TNF-a and IL-1bT levels were found higher in the aged subjects, whereas TNF-a levels remained similar.

A more recent study [6] evaluated the differences in blood concentrations of hsCRP, IL-6 and TNF-α between 110 healthy subjects, divided into 5 groups according to age (21–30, 31–40, 41–50, 51–60, and 61–70 years). Higher values of CRP and IL-6 were found in older individuals.

Despite these existing studies published in the literature, there are limitations when trying to understand the correlation between aging and inflammatory markers independently from comorbidities. The major aspects to consider in these studies are the small numbers of volunteers included which are not representative of the general population, and the lack of substantial data of people over 80 years old. In addition, currently there are no longitudinal studies that show how the levels of inflammation biomarkers changes over the years in otherwise healthy subjects.

The present paper reports correlations between healthy aging and systemic inflammation on a large US representative sample of aging residents.

## Methods

### Data source

Cross-sectional data were extracted from the publicly available 2016 Health and Retirement Survey (HRS), a nationally representative study of older adults in the United States (US) that includes in-depth interviews assessing respondents’ demographics, physical health, family, and financial status [7]. The first interviews were conducted in 1992, with follow-up surveys every 2 years. During the 2016 cycle, HRS collected a venous blood sample from a subset of participants [8], that consented to a blood draw and were measured for high sensitivity C-reactive protein (hs-CRP), Interleukin (IL)-6, IL-10, IL-1RA, soluble tumor necrosis factor receptor (sTNFR-1) and transforming growth factor beta 1 (TGF-β1). Details on sample collection and processing are provided by HRS [9].

The HRS is sponsored by the National Institute on Aging (grant number NIA U01AG009740) to the University of Michigan. As data were publicly available, IRB approval was not required for this analysis.

### Study population

There were 20, 912 participants who responded to the 2016 survey. Only those with a venous blood draw were included for analysis (n = 9934). Those who were missing some of the required measurements, survey weighting information, or were age ineligible for a venous blood draw (n = 746; 7.5%) were excluded, for a final sample of 9,188 participants.

### Variables of interest

Demographics and self-reported health conditions were extracted from the survey. Participants were asked if a doctor had ever told them they had high blood pressure, diabetes, cancer (excluding non-melanoma skin cancer), lung disease (including chronic bronchitis and emphysema), a heart condition (including heart attack, coronary heart disease, angina, congestive heart failure, and other heart problems), a stroke, Alzheimer’s disease or other dementia, or arthritis. An index was created for the number of health conditions a respondent reported.

Based on guidance from HRS [10], extremely high values of inflammatory markers were recoded as missing (7 observations for IL-6, <5 observations for all other markers). Inflammatory markers were natural log (ln)-transformed when used as continuous variables and were also dichotomized into the top 10% and bottom 90% of values.

Follow up information was obtained through the 2018 interview. Participants were recorded as deceased if a date of death was provided either through an exit interview, or by a spouse or partner. For the remaining, the last known date the participant was alive was recorded as the most recent of the last core interview of either the respondent or their spouse/partner reporting the participant to be alive, or the last date on which researchers talked to the respondent or someone with direct knowledge of the respondent.

### Statistical analysis

In order to account for the complex sampling strategy of HRS, all analyses incorporated the survey design variables provided by HRS. Inflammatory markers were plotted according to age, and unadjusted linear regressions were conducted to assess the correlation. Multivariable linear regressions were used to assess the independent correlation between age and inflammation, adjusting for sex and the number of health conditions. A separate set of models were run to assess the correlation between inflammatory markers and each health condition individually. A subset analysis was conducted on the subset of participants who reported no health conditions. Cox-proportional hazards models were used to assess the independent correlation of inflammatory markers (as both continuous and dichotomous variables) with time to death. Interactions between inflammatory markers and age were included in the models. Analyses were conducted using SAS software, version 9.4 (SAS Institute, Cary, NC) and R version 4.0.5/R studio version 1.2.1335.

## Results

There were 9,188 participants included representative of 83,939,225 nationally. Participants were 68.7 years old on average, majority female (54.2%), white/Caucasian (82.9%), non-Hispanic (90.9%), and born in the US (89.6%). Nearly 87% had at least a high school diploma/GED and 63.0% had a spouse or partner. Almost 36% of participants self-reported at least 3 health conditions, with the most common being arthritis (60.4%), high blood pressure (59.0%), heart condition (25.2%) and diabetes (24.9%) (Table 1).

**Table 1:** Description of the study cohort.

Variable	Study Cohort (n = 9,188) n (Weighted %)
age, years (SE: standard error), mean	68.7 (0.3)
age, years (IQR: interquartile range), median	66.2 (60.7–73.9)
Sex	
Male	3885 (45.8)
Female	5303 (54.2)
Race	
White/Caucasian	6767 (82.9)
Black/African American	1637 (10.4)
Other	758 (6.5)
Missing/Unknown	26 (0.2)
Ethnicity	
Non-Hispanic	7888 (90.9)
Hispanic	1289 (9.0)
Missing/Unknown	11 (0.1)
Country of Birth	
United States	7951 (89.6)
Outside of the United States	1233 (10.4)
Missing/Unknown	4 (0.0)
Highest Level of Education	
< High School	1577 (13.3)
GED/High School Diploma	4735 (50.5)
Some college/Associate’s Degree	666 (7.6)
≥4 year college	2234 (28.6)
Marital Status	
Married/Partnered	5730 (63.0)
Separated/Divorced	1293 (14.7)
Widowed	1725 (16.5)
Never Married	430 (5.7)
Unmarried, NOS	10 (0.1)
Health Conditions	
High Blood Pressure	5925 (59.0)
Diabetes	2613 (24.9)
Cancer	1546 (16.2)
Lung Disease	1090 (11.4)
Heart Condition	2502 (25.2)
Stroke	720 (7.1)
Dementia/Alzheimer’s	212 (3.0)
Arthritis	5981 (60.4)
Number of Health Conditions	
0	971 (13.6)
1	1950 (23.6)
2	2543 (27.2)
≥3	3724 (35.6)

**Fig 1:**
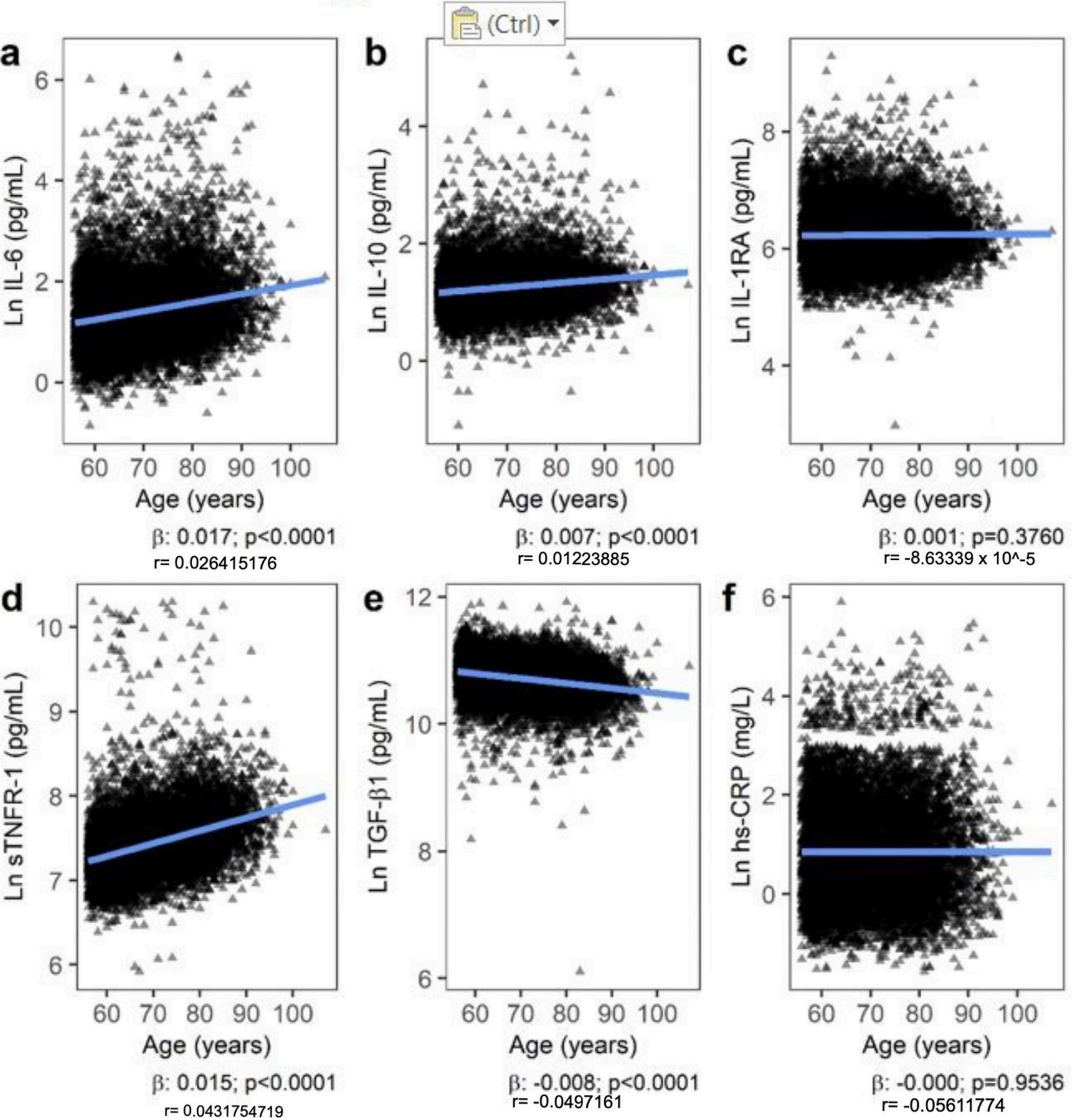
Bivariate association between age and inflammatory markers (log-adjusted) a) IL-6; b) IL-10; c) IL-1RA; d) sTNFR-1; e) TGF-β1; f) hs-CRP.

Before adjustment, there was a significant positive correlation between age and IL-6 (β = 0.017; p<0.0001), IL-10 (β = 0.007; p<0.0001), and sTNFR-1 (β = 0.015; p<0.0001), and a significant inverse correlation with TGF-β1 (β = -0.008; p<0.0001) (Fig 1).

After adjusting for sex and the number of health conditions there remained a significant positive correlation between age and ln IL-6 (β_adj_: 0.012, 95% CI: 0.009–0.014), ln IL-10 (β_adj_: 0.005, 95% CI: 0.003–0.007), and ln sTNFR-1 (β_adj_: 0.012, 95% CI: 0.011–0.013). There was a significant inverse correlation between age and ln IL-1RA (β_adj_: -0.004, 95% CI: -0.005- -0.002), ln TGF-β1 (β_adj_: -0.007, 95% CI: -0.008- -0.006), and ln hs-CRP (β_adj_: -0.007, 95% CI: -0.010- -0.003) (Table 2).

**Table 2:** Factors independently associated with inflammatory markers.

Variable	Ln IL-6 (n = 9083) Β_adj_ (95% CI)	Ln IL-10 (n = 9088) Β_adj_ (95% CI)	Ln IL-1RA (n = 9088) Β_adj_ (95% CI)	Ln sTNFR-1 (n = 9088) Β_adj_ (95% CI)	Ln TGF-β1 (n = 9088) Β_adj_ (95% CI)	Ln hs-CRP (n = 9106) Β_adj_ (95% CI)
Age, years	**0.012 (0.009–0.014)**	**0.005 (0.003–0.007)**	**-0.004 (-0.005- -0.002)**	**0.012 (0.011–0.013)**	**-0.007 (-0.008- -0.006)**	**-0.007 (-0.010- -0.003)**
Sex						
Female vs. Male	**-0.048 (-0.078- -0.017)**	**-0.049 (-0.067- -0.031)**	**0.069 (0.038–0.101)**	-0.004 (-0.019–0.011)	**0.033 (0.017–0.048)**	**0.142 (0.092–0.193)**
Number of Health Conditions						
0	**-0.444 (-0.522- -0.367)**	**-0.166 (-0.206- -0.126)**	**-0.354 (-0.406- -0.303)**	**-0.278 (-0.308- -0.249)**	**0.051 (0.025–0.077)**	**-0.565 (-0.652- -0.478)**
1	**-0.344 (-0.391- -0.297)**	**-0.130 (-0.159- -0.100)**	**-0.237 (-0.269- -0.204)**	**-0.217 (-0.243- -0.190)**	**0.052 (0.030–0.073)**	**-0.352 (-0.424- -0.280)**
2	**-0.195 (-0.254- -0.135)**	**-0.072 (-0.101- -0.042)**	**-0.136 (-0.168- -0.102)**	**-0.148 (-0.177- -0.118)**	**0.040 (0.022–0.058)**	**-0.212 (-0.289- -0.135)**
≥3	0.0 (ref)	0.0 (ref)	0.0 (ref)	0.0 (ref)	0.0 (ref)	0.0 (ref)

Adjusted for all listed variables; Bold indicates statistical significance

Among the subset of participants who reported none of the included health conditions (n = 971), there remained an independent correlation of age with ln IL-6 (β_adj_:0.023, 95% CI: 0.013–0.032) and ln sTNFR-1 (β_adj_:0.012, 95% CI: 0.009–0.015). There was a significant inverse correlation between age and ln TGF-β1 (β_adj_: -0.004, 95% CI: -0.007- -0.001). Although not significant, there was a positive correlation between age and ln IL-1RA and ln hs-CRP (S1 Fig).

There was a significant positive correlation between individual health conditions and all inflammatory markers, except ln TGF-β1, which was significantly inversely associated with diabetes, cancer, and heart disease (S1 Table).

The median (IQR) follow up for the sample was 23.0 (20.0–25.0) months. After adjusting for age, sex, and number of reported health conditions, there was a statistically significant correlation between increased ln IL-6, ln IL-10, ln sTNFR-1, and ln hs-CRP with death. Results were similar when comparing those with the highest 10% of values to those in the bottom 90% (Table 3 and S2 Fig).

**Table 3:** Independent correlation of inflammatory markers with survival a) log-adjusted and b) comparison of the top 10% of values to the bottom 90%.

a.		b.	
Inflammatory Marker (Log-adjusted)	Adjusted HR (Hazard Ratio) (95% CI)*	Inflammatory Marker (Top 10% Yes vs. No)	Adjusted HR (95% CI)*
IL-6	**1.47 (1.28–1.69)**	IL-6 (>10.2 pg/mL)	**2.10 (1.48–2.96)**
IL-10	**1.86 (1.50–2.30)**	IL-10 (>5.5 pg/mL)	**2.25 (1.64–3.10)**
IL-1RA	1.30 (0.94–1.79)	IL-1RA (>980 pg/mL)	1.05 (0.64–1.74)
sTNFR-1	**3.03 (2.42–3.79)**	sTNFR-1 (>2632.8 pg/mL)	**2.50 (1.73–3.61)**
TGF-β1	0.79 (0.46–1.34)	TGF-β1 (>66,234 pg/mL)	1.73 (0.94–3.20)
hs-CRP	**1.47 (1.28–1.69)**	hs-CRP (>8.7 mg/L)	**2.76 (1.90–3.99)**

*Adjusted for age, sex, and number of health conditions; bold indicates statistical significance

Survival models were stratified by age (Table 4). When considering inflammatory markers as continuous measures, there was a larger correlation between elevated inflammatory markers and death in younger than older participants for IL-6 (p-value [interaction] = 0.0351) and hs-CRP (p-value [interaction] = 0.0141). The interaction between age and TGF-β1 was also statistically significant (p = 0.0055). When inflammatory markers were considered as dichotomous (≤ or > the 90^th^ percentile of values), the correlation between elevated inflammation and death was attenuated by increasing age for IL-6 (p-value [interaction] = 0.0007), IL-10 (p-value[interaction] = 0.0168), and hs-CRP (p-value[interaction] = 0.0045). This trend was also true for IL-1RA (p-value[interaction] = 0.0682). The correlation between TGF-β1 and death grew larger with age (p-value[interaction] = 0.0018).

**Table 4:** Independent correlation of inflammatory markers with survival, stratified by age group a) log-adjusted and b) comparison of the top 10% of values to the bottom 90%.

a.	Adjusted HR (95% CI)*				
Inflammatory Marker (Log Adjusted)	56–65 years	66–75 years	76–85 years	≥85 years	Interaction p-value
IL-6	**2.39 (1.41–4.05)**	**1.62 (1.27–2.07)**	**1.40 (1.23–1.59)**	1.20 (0.95–1.52)	0.0351
IL-10	**2.91 (1.40–6.05)**	**2.22 (1.41–3.50)**	**2.19 (1.74–2.74)**	1.25 (0.79–1.98)	0.1379
IL-1RA	1.33 (0.47–3.80)	1.67 (0.90–3.11)	1.19 (0.81–1.74)	0.98 (0.55–1.74)	0.2058
sTNFr-1	**3.38 (2.28–4.72)**	**3.07 (2.12–4.45)**	**2.69 (1.87–3.85)**	**4.29 (1.92–9.58)**	0.7176
TGF-β1	**0.06 (0.02–0.15)**	1.56 (0.73–3.33)	0.91 (0.55–1.49)	1.68 (0.45–6.33)	0.0055
hs-CRP	**1.97 (1.18–3.27)**	**1.76 (1.41–2.19)**	**1.50 (1.28–1.75)**	1.23 (0.98–1.53)	0.0141
**b.**	Adjusted HR (95% CI)*				
Inflammatory Marker (Top 10% Yes vs. No)	56–65 years	66–75 years	76–85 years	≥85 years	
IL-6 (>10.2 pg/mL)	**4.79 (1.88–12.23)**	**3.62 (1.83–7.17)**	**2.33 (1.62–3.36)**	1.00 (0.55–1.84)	0.0007
IL-10 (>5.5 pg/mL)	**4.34 (1.77–10.45)**	**3.50 (1.62–7.51)**	**2.34 (1.42–3.87)**	0.98 (0.42–2.29)	0.0168
IL-1RA (>980 pg/mL)	1.33 (0.44–4.02)	1.24 (0.52–2.95)	1.00 (0.52–1.91)	0.53 (0.17–1.64)	0.0682
sTNFR-1 (>2632.8 pg/mL)	**7.51 (3.09–18.29)**	**3.41 (1.68–6.91)**	**1.79 (1.16–2.76)**	**2.44 (1.23–4.82)**	0.1769
TGF-β1 (>66,234 pg/mL)	0.30 (0.08–1.13)	1.61 (0.72–3.64)	1.73 (0.66–4.54)	**3.68 (1.08–12.55)**	0.0018
hs-CRP (>8.7 mg/L)	**4.85 (1.64–14.30)**	**4.42 (2.38–8.23)**	**2.22 (1.37–3.58)**	1.65 (0.88–3.08)	0.0045

*Adjusted for age, sex, and number of health conditions; bold indicates statistical significance

## Discussion

Through a large, nationally representative, US aging population with a wide age range including those 90 years and older, we highlight the existence of a correlation between serum biomarkers of inflammation and aging. This can be observed not only in the whole population included in this analysis, but also in the smaller subset of subjects (approximately 900 subjects) who reported no comorbidities or health conditions. This confirms the existence of a presence of low-grade inflammation in aging, even in healthy elders. Additionally, the results presented show that there is a linear correlation between four inflammatory markers (IL-6, IL-10, sTNFR-1, hs-CRP) and overall mortality. The correlation was present even after adjustment for comorbidities and its strength changes with age. In fact, it is not significant in the subgroup of individuals age 85 years old and above. Although a probable reason for this change in correlation with age could be the presence of competitive risk for chronic conditions and death at earlier ages, this observation should be the basis for further ad hoc studies.

In the present analysis, we observed a statistical interaction between some of the inflammatory markers and age on mortality, although it is not possible from the available data to study if such an interaction reflects an underlying biological mechanism involving specific inflammatory pathways. The public health implications of these results are complex for various reasons. First of all, it is unclear at the moment what the optimal range of inflammatory markers is for healthy aging and longevity, and more generally if there is a safe threshold. Although we could propose to measure multiple inflammatory markers to determine a profile of healthy aging, it is still impractical to set up a study that could identify values correlated with an increased mortality risk to the subject. For example, hs-CRP is a good marker of systemic inflammation, however it could be episodically elevated because of acute inflammation. Such a study would thus require repeated measures and a more complex design than the analysis we present here. Previously, only a few studies addressed the relationship with serum biomarkers of inflammation and mortality, but none of them had the breadth of our analysis. One cohort [11] in the UK has shown that increased inflammatory markers are predictors of one-year mortality. because they included patients drawn from health care settings, such as their general practitionerit is difficult to assess if the data represent what occurs in the general population of healthy elders. In 2019 a cohort study conducted in Brazil [12] highlights the correlation between hsCRP level and overall mortality, independently of lifestyle and clinical variables. It should be considered that this study included a much smaller cohort than the one involved in the present study, is representative of the Brazilian population but not necessarily of the US population and had an upper age limit to 74 years. There are also a few published studies on the relationship between hs-CRP and IL-10 with cardiac mortality. Recently a cohort study in 2022 of 102,337 patients with suspected heart attacks was conducted to assess if higher hsCRP levels were correlated with higher mortality r. A dose-response relationship was noted, with hazard ratio of 1.32 for those with hsCRP 2.0 to 4.9 mg/L, and 2.00 for those with hsCRP 10 to 15 mg/L. [13] Additionally it is known [14] that inflammation plays a major part in the development of atherosclerosis and coronary heart disease and therefore the levels of these markers could be an expression of the underlying inflammatory process affecting the cardiovascular system. Our findings of an inverse correlation between hs–CRP and age after adjusting for comorbidities (β_adj_: -0.007, 95% CI: -0.010- -0.003) were not confirmed in the subgroup of otherwise healthy individuals. The inverse association of hs-CRP with age is inconsistent with prior studies and is potentially an area to further study. A 2019 study analyzed 110 individuals divided into 5 groups according to their age and reported a positive correlation between hsCRP and aging (r = 0.369), with the highest values of hsCRP for the age range 51–60 years [6]. In another study conducted in 2018 among 6060 Chinese healthy subjects, serum levels of hs-CRP were significantly increased with aging (*P* < 0.05), particularly in those with age over 45-year-old [15]. We do observe, however, a statistically significant association between hs-CRP and overall mortality, an association that decreases in strength at older ages, thus suggesting that the relationship between hs-CRP, age and mortality could be more complex than we hypothesize.

A correlation between sTNFR-1 levels and stroke has also been identified in the present study. There have been debates over the exact mechanism behind this phenomenon, however studies [16] have established that anti-TNF treatments have reduced both the risk and prevalence of stroke [17]. It is possible that sTNFR-1, similar to hs-CRP, is a marker of the presence of an underlying local vascular inflammation [18].

In addition, the lack of definition regarding the optimal range of inflammatory markers makes it difficult to determine the effectiveness of treatment targeting inflammation in decreasing and delaying mortality. Despite this, it must be noted that treatments targeting inflammation have been shown to be beneficial, the 2017 10,000-patient CANTOS study [19] provided evidence that anti-inflammatory therapy targeting IL-1B with canakinumab at a dose of 150mg every 3 months led to lo significantly lower rate of cardiovascular events as compared to a placebo. In this study however, the population analyzed was not a sample of the general population, but rather had a history of myocardial infarction and a blood level of high-sensitivity C-reactive protein of 2 mg or more per liter despite the use of aggressive secondary prevention strategies.

Our study has some limitations; we don’t have complete information on obesity, smoking status or other health condition outside the ones outlined previously, because of the nature of the dataset used for the analysis. Prior studies [20] have noted that inflammatory biomarkers are elevated in obese individuals. In this case, we are unsure how many individuals considered in this study were obese, and how that would potentially affect the results. Additionally, we were limited in the selection of inflammatory markers to analyze considering the study was based on an existing database created by the HRS which pre-selected the cytokines. This also limited us in performing any additional tests on these subjects. Also, it is important to note that most of the studies cited, including ours, rely on one point measure of inflammatory markers to show their correlation with mortality. Additional repeated measures could increase the predictive value by showing seasonal and daily variations of these biomarkers. With the existing available data, we cannot predict whether repeated measures would increase the predictive value of the biomarker, or one random measure is already reflecting a chronic condition of inflammation over a lifetime.

In conclusion, we show here, using a large representative U.S. dataset, that aging is associated with increased levels of several inflammatory markers, and that this correlation is present over a large range of ages, in subjects otherwise self-reported as healthy. We also highlight the existence of a correlation between inflammatory markers and overall mortality. Future studies should address a possible threshold of systemic inflammation where mortality significantly increases, as well as explore the effectiveness of anti-inflammatory treatments on morbidity and mortality in healthy aging subjects along with studying more in depth the inverse correlation between hs-CRP and aging.

## Supporting information

S1 Fig Distribution of log-adjusted inflammatory markers by age group and number of health conditions a) IL-6; b) IL-10; c) IL-1RA; d) sTNFR-1; e)TGF-β1; f) hs-CRP.
(TIF)

S2 Fig Cox Regression Survival Function Analysis of Inflammatory markers a) IL-6; b) IL-10; c) IL-1RA; d) sTNFR-1; e) TGF-B1; f) hs-CRP divided into top 10% values (B) and bottom 90% values (A).
(TIF)

S1 Table Health conditions independently associated with inflammatory markers. (TIF)
