# Supplementary figures and images for: Blood inflammatory markers and mortality in the US population: A Health and Retirement Survey (HRS) analysis
Source: PLoS One. 2023 Oct 16;18(10):e0293027. doi: 10.1371/journal.pone.0293027 (PMC10578595; doi:10.1371/journal.pone.0293027)

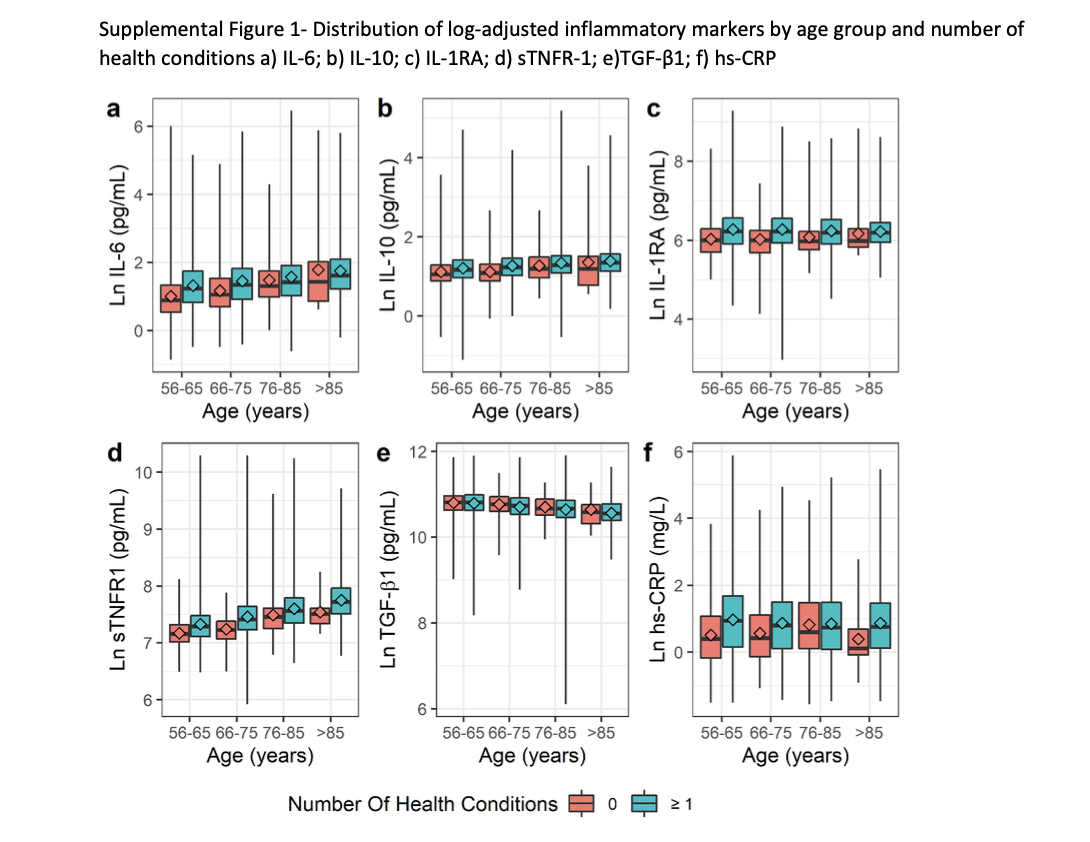

Supplement: S1 Fig — Distribution of log-adjusted inflammatory markers by age group and number of health conditions a) IL-6; b) IL-10; c) IL-1RA; d) sTNFR-1; e)TGF-β1; f) hs-CRP. (TIF) [file pone.0293027.s001.tif]

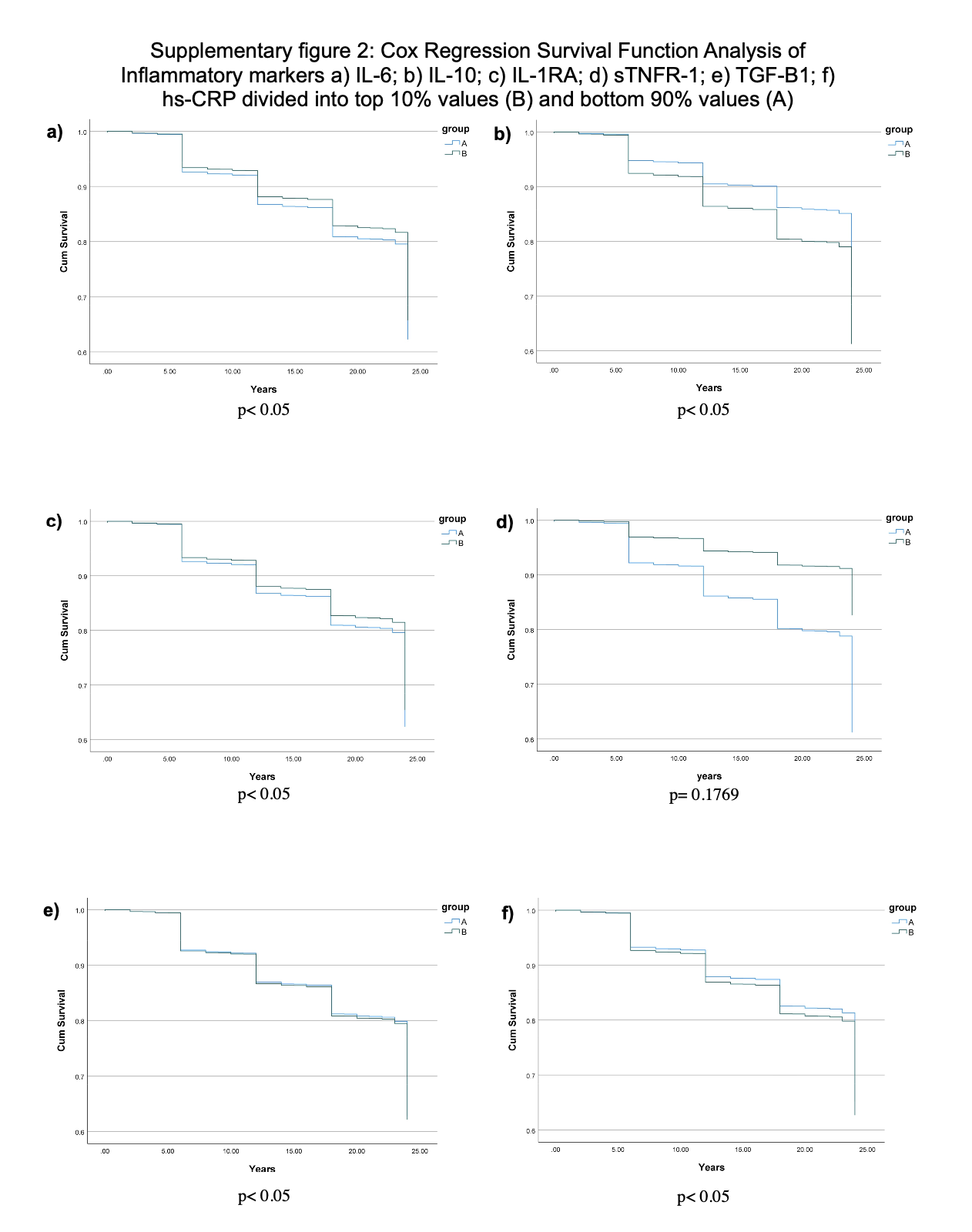

Supplement: S2 Fig — Cox Regression Survival Function Analysis of Inflammatory markers a) IL-6; b) IL-10; c) IL-1RA; d) sTNFR-1; e) TGF-B1; f) hs-CRP divided into top 10% values (B) and bottom 90% values (A). (TIF) [file pone.0293027.s002.tif]

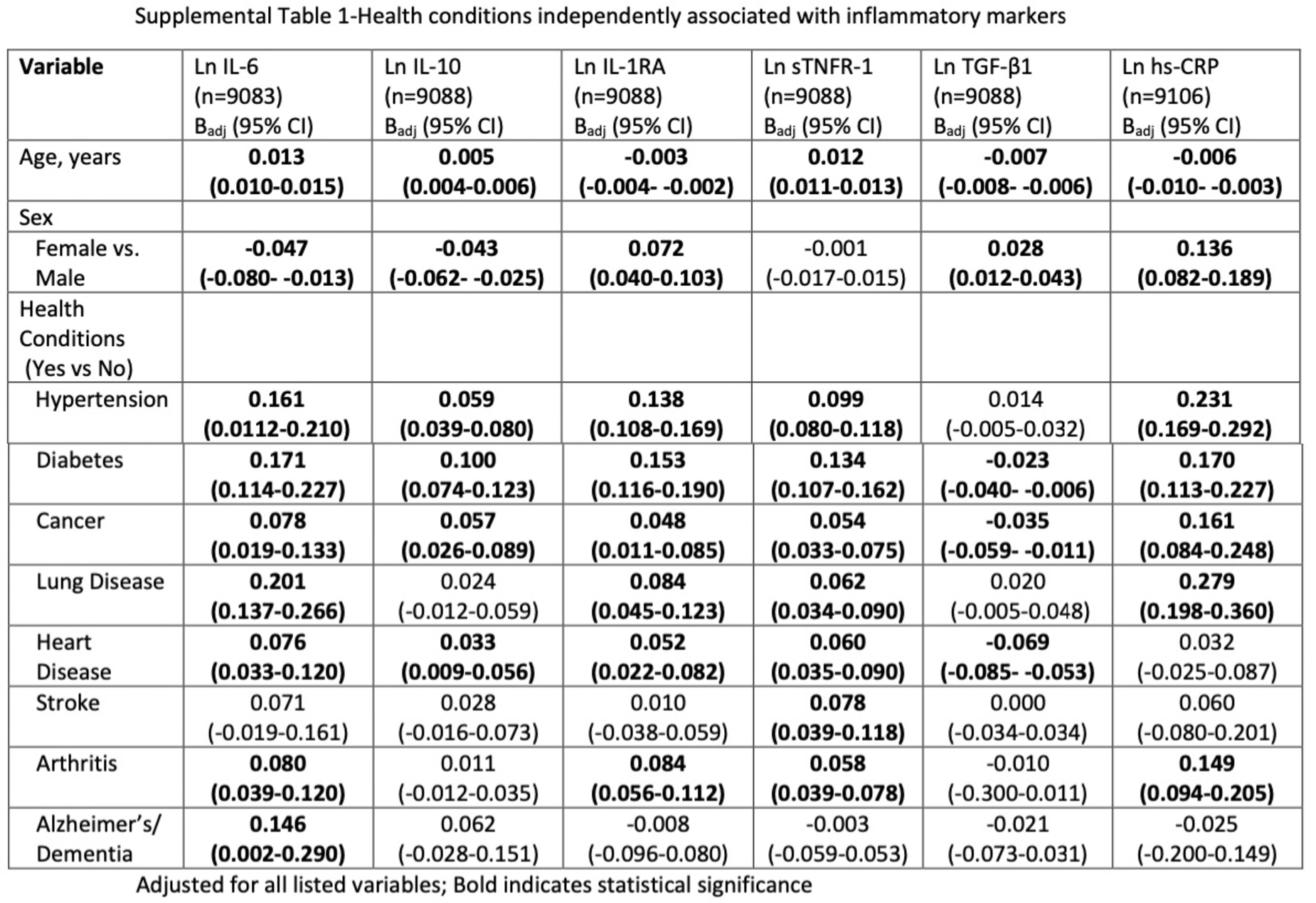

Supplement: S1 Table — (TIF) [file pone.0293027.s003.tif]
